# Portrayals of branded soft drinks in popular American movies: a content analysis

**DOI:** 10.1186/1479-5868-3-4

**Published:** 2006-03-09

**Authors:** Diana Cassady, Marilyn Townsend, Robert A Bell, Mitchell Watnik

**Affiliations:** 1 Department of Public Health Sciences, University of California, PHS: Research and Outreach Programs, One Shields Avenue, Davis, CA 95616, USA; 2 Department of Nutrition, University of California, One Shields Avenue, Davis, CA, USA; 3 Department of Communication, University of California, One Shields Avenue, Davis, CA, USA; 4 Department of Statistics, California State University East Bay, Hayward, CA, USA

## Abstract

**Background:**

This study examines the portrayals of soft drinks in popular American movies as a potential vehicle for global marketing and an indicator of covert product placement.

**Methods:**

We conducted a content analysis of America's top-ten grossing films from 1991 through 2000 that included portrayals of beverages (95 movies total). Coding reliabilities were assessed with Cohen's kappa, and exceeded 0.80. If there was at least one instance of branding for a beverage, the film was considered having branded beverages. Fisher's exact test was used to determine if soft drink portrayals were related to audience rating or genre. Data on the amount of time soft drinks appeared onscreen was log transformed to satisfy the assumption of normality, and analyzed using a repeated measures ANOVA model. McNemar's test of agreement was used to test whether branded soft drinks are as likely to appear or to be actor-endorsed compared to other branded beverages.

**Results:**

Rating was not associated with portrayals of branded soft drinks, but comedies were most likely to include a branded soft drink (p = 0.0136). Branded soft drinks appeared more commonly than other branded non-alcoholic beverages (p = 0.0001), branded beer (p = 0.0004), and other branded alcoholic beverages (p = 0.0006). Actors consumed branded soft drinks in five times the number of movies compared to their consumption of other branded non-alcoholic beverages (p = 0.0126). About half the revenue from the films with portrayals of branded soft drinks come from film sales outside the U.S.

**Conclusion:**

The frequent appearance of branded soft drinks provides indirect evidence that product placement is a common practice for American-produced films shown in the U.S. and other countries.

## Background

Soft drinks are suspected to contribute to the global obesity crisis. For instance, the World Health Organization concluded that there is probable scientific evidence to support an association between soft drink consumption and obesity[1]. It reported that the *marketing *of soft drinks contributes to the global obesity crisis.

Soft drink consumption has skyrocketed. Since 1997, per capita consumption of soft drinks has increased by double digits each year in the largest soft drink markets: the United States, Brazil, Japan, Mexico, Germany, and China[2]. Annual consumption in the U.S. is highest at 161 liters per capita[2]. Between 56% and 85% of American school-age children drink at least one soft drink daily, with the highest amounts ingested by adolescent males. Of this group, 20% consume 4 or more servings daily[3].

A 12-oz soft drink consumed daily has been associated with a 0.18 kg/m^2 ^(BMI units) increase in a child's body mass index and a 60% increase in risk of obesity[4]. Sugar-sweetened soft drinks are associated with obesity, probably because over consumption is a particular problem when energy is ingested in liquid form[5] and because these drinks represent the leading source of added sugar in the diet[4].

In the United Kingdom and the United States, government officials and health experts have called for limits on the marketing of "junk food" including soft drinks[6,7]. Recommendations for marketing limits are based, in part, on studies of food advertising on television[8-11]. Especially during children's programming, televised food advertising typically "sells" the idea by associating the product with joy and happiness for the child[12]. Such advertisements have typically promoted foods high in fat, sugar, and salt, and ignored fruits, vegetables, and complex carbohydrate sources[8-10]. For instance, during Saturday morning children's programming in the U.S., 5.6% of ads were for soft drinks, but only 1.6% were for milk and 0% were for fruit juice[8]. Studies on advertising exposure and eating choices have focused on children, and indicate that children tend to seek out foods that are heavily advertised[10,13,14].

The presence of branded products in movies is often a form of paid advertising[15,16] yet no studies examine portrayals of branded soft drinks in popular movies. Product placement occurs when an advertiser pays a movie studio to put its product in a movie. Placement fees paid by advertisers range from $5,000 to $100,000, and are sometimes waved in exchange for promotional tie-ins where advertisers feature the movie as part of their regular product marketing. The movie industry charges more when the branded product is consumed by an actor[17,18].

Product placements in movies have been used to sell fast food, cars, cigarettes, beer, and soft drinks, and are negotiated by third-party product placement companies. An exception is Coca Cola which has its own product placement office in Hollywood[16]. Unlike explicit advertising on television or billboards, product placement in movies is covert. According to one beverage company executive, "When a product is embedded in the content of a movie or a show, it can carry increased credibility with our target audience[19]."

The focus of this study is on movie portrayals, not on their effects. Furthermore, we assume that many appearances of soft drinks in movies are paid, but the proprietary nature of product placement makes it impossible to verify payment. Nevertheless, there is cause for concern about the possible effects of implicit advertising in movies. Movies provide vicarious influences for viewers in the form of abundant symbolic modeling[20]. According to Bandura, modeling influences were confined to the behavior exhibited in one's community until the advent of mass media. Now symbolic modeling is provided by film, television and other forms of mass media and enables people to transcend the bounds of their immediate social environment[21]. Health behaviors repeatedly shown in movies become widely accepted as normative and might include frequent consumption of soft drinks as a replacement for milk by children and adolescents or consumption of extra calories by physically inactive children, adolescents, and adults. If attractive, physically fit role models viewed on the screen demonstrate a preference for drinking soft drinks, then viewers may want to demonstrate the same preference for soft drinks. No negative consequences of regular consumption of soft drinks are portrayed on screen. This implies that routinely consuming soft drinks is a desirable part of an athletic, healthy lifestyle, and is the more acceptable beverage of choice.

This study examines the use of popular American movies as a potential vehicle for advertising soft drinks by investigating several issues. First, we examined the prevalence of branded soft drinks. To better understand these prevalence data, we compared the frequency with which branded soft drinks appeared with the frequencies with which other kinds of branded and nonbranded beverages appeared in these movies, including alcohol. Second, we examined the extent to which branded soft drinks were shown being consumed, relative to other drinks, with endorsement conceived of as a product endorsement. Third, we sought to understand better the manner in which these soft drinks were portrayed by comparing their duration on the screen with the length of presentation of other beverage types; by examining whether they were more likely to appear in movies made for younger audiences (P and PG rated movies); and by examining their use as props as a function of the era in which the film was situated. Fourth, we examined the extent to which portrayals of soft drinks in these movies had global reach, using international box office revenues as a proxy for international exposure

## Methods

### Film sample

The sample of films consisted of America's top-ten grossing movies each year from 1991 through 2000. We selected the top-ten grossing films in an effort to analyze the movies most likely to have a wide range of appeal to the general American public. This analysis examines the portrayal of soft drinks and other beverages in the dataset, and is part of a larger study of portrayals of food and physical activity in popular American films[22]. Out of the 100 movies in the sample, five were excluded because they had no portrayals of beverages. The final sample included 95 films.

### Content analysis

A beverage was considered eligible for inclusion in the study if it was ingested by a human character (real or animated); was being ordered, bought, or in anyway acquired by a human character; or was being held, prepared, or in immediate proximity to a human character. Following the method of Sargent et al. in their study of tobacco brand portrayals in popular movies,[23] each appearance of a beverage was coded as a brand appearance or as an actor endorsement. Brand appearance was defined as the presence of a branded beverage. An appearance of a beverage was coded as actor-endorsed if an actor consumed the branded beverage.

Two undergraduates were trained by the investigators to apply the coding instrument and procedures. Coders spent 5–10 hours watching each film and recording all portrayals of food and beverages and intentional fitness activity. One-third of films were double-coded to assess the reliability (inter-judge agreement), and one of the authors arbitrated coding disagreements. Coding reliabilities were assessed with Cohen's kappa. Kappa scores were 0.81 for branding and for 0.91 for ingestion of beverages, resulting in "almost perfect" reliabilities for kappa scores according to the categorizations developed by Landis and Koch[24].

### Film revenues and genres

We obtained movie revenues from . This web site uses several entertainment industry and trade sources to estimate revenues earned from worldwide distribution of films shown in movie theaters, but not from video or DVD rentals or sales. Movies were categorized by audience rating, genre, and time period represented in the film. Genres were identified using the first listing under "genre" on the Internet Movie Database , and included categories such as "action", "drama", "children's movie", etc. Each movie's time period setting was taken from the film summary on the Internet Movie Database or by student coders who watched the movies for indications of time period setting. Time period setting was defined by changes in soft drink production and consumption in the United States. These time periods include before 1890 when soft drinks were only available in drug stores and restaurants; 1890–1960 when soft drink sales were either not recorded or remained relatively low at about 10 gallons per capita (37.85 liters per capita); 1960–2000 when soft drink consumption began increasing rapidly; and, the future (after 2000)[25]. In the rare instances when movies spanned time periods, the predominate time setting was used.

### Statistical analyses

Research questions concerning the absolute and relative frequency of portrayals and movie revenues were addressed using basic descriptive statistics (frequencies, percentages, rates). All statistical tests analyze the data on a film by film basis, regardless of the duration of the portrayal of the beverage. If there is at least one branding for a beverage, the film is considered having branded beverages. For instance, one branded appearance of a soft drink places the film in the branded category, even if all other appearances of soft drinks are unbranded. Fisher's exact test was used to determine if soft drink portrayals were statistically related to audience rating or genre. Data on the amount of time soft drinks appeared onscreen was log transformed to satisfy normality, and analyzed using a repeated measures ANOVA model where brand (yes/no) served as the independent variable and number of seconds onscreen served as the repeated measure. Results were back transformed and presented as the median number of seconds[26]. McNemar's test of agreement was used to test whether branded soft drinks are as likely to appear or to be actor-endorsed compared to other branded beverages.

## Results

Twenty-one percent of the movies were rated G for general audience or PG for "parental guidance" and so were appropriate for all ages (Table 1). The majority of movies were action (32 of 95 [34%]) or comedies (24 of 95 [25%]), although all genres were represented. Thirteen percent of the movies were children's movies, such as animation, fantasy, or family. The majority (80 of 95 [85%]) were set between 1960–2000, the period when soft drink consumption was rising rapidly in the U.S.

**Table 1:** Characteristics of 95 movies, 1991–2000

MPAA Rating *	
G	3 (3%)
PG	17 (18%)
PG-13	44 (46%)
R	31 (33%)
**Genre**	
Action Adventure	32 (34%)
Children/Family/Animation	12 (13%)
Comedy	24 (25%)
Drama	13 (14%)
Horror	4 (4%)
Mystery/Thriller/Crime	7 (7%)
Science Fiction	3 (3%)
**Time-period setting**	
Before 1890	6 (6%)
1890–1959	6 (6%)
1960–2000	80 (85%)
Future	3 (3%)
**Portrayed Soft Drinks**	
Yes	43 (45%)
No	52 (55%)
**Portrayed Branded Soft Drinks**	
Yes	31 (33%)
No	64 (67%)

*The U.S. has a voluntary system of movie ratings that helps parents prevent their children from seeing movies that are inappropriate. G is for a general audience, with all ages admitted. PG recommends parental guidance, and some material may not be suitable for children. PG-13 means "parents are strongly cautioned" and some material may be inappropriate for children under 13. R is restricted for viewers; anyone 17 years or younger requires an accompanying parent or guardian.

### The prevalence of branded soft drinks

**Figure 1:**
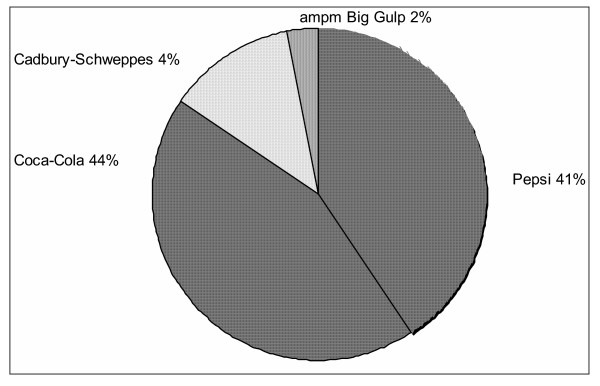
Distribution of 31 Movies by Soft Drink Company.

Forty-five percent of movies (43 of 95) included at least one depiction of soft drinks, and the majority of these movies with soft drinks showed at least one portrayal of a branded soft drink (31 of 45 [72%]). Brands from four companies were depicted in these 31 movies: the Coca-Cola Company's brands Coke, Diet Coke, and Sprite; Pepsi-Cola Company's brands Pepsi and Diet Pepsi; Cadbury Schweppes' brands Dr. Pepper and 7-Up; and ampm's Big Gulp. Of these four companies, Pepsi and Coca-Cola accounted for 85% (26 of 31) of the movies with branded soft drinks (Figure 1). *Wayne's World *was the only movie that included brands from competing companies (Pepsi and Dr. Pepper). Three other movies showed more than one brand of soft drink, but the brands were owned by the same company.

There was no relationship between movie rating and portrayals of branded soft drinks. Some genres were more likely to portray branded soft drinks (p = 0.0136). For instance, comedies were more likely to include a branded soft drink compared to horror or drama movies. Examples of youth-oriented comedies that included the most depictions of soft drinks include *Home Alone 2*, *There's Something About Mary*, and *Wayne's World*.

There was no statistically significant difference between the amount of time branded and unbranded soft drinks appeared on screen. The median time unbranded soft drinks appeared was 38.5 seconds (95% CI 14.6–94.2), while the median time branded soft drinks appeared onscreen was 62.02 seconds (95% CI 35.5–111.4). While these differences are not statistically significant, they are in the anticipated direction: Branded soft drinks were on screen over 60% longer than unbranded soft drinks.

### Portrayals of branded soft drinks compared to other branded beverages

We also examined whether there was a difference in brand appearance and actor endorsement for branded soft drinks compared to other branded beverages. For this analysis branded soft drinks were grouped into one category and were compared to three other categories of branded beverages: other non-alcoholic drinks (e.g., water, juice, milk, coffee, tea); beer; and other alcoholic drinks (e.g., wine, liquor, mixed drinks).

Branded soft drinks appeared in a significantly greater proportion of movies compared to the other 3 beverage categories (Table 2). Branded soft drinks appeared in 15 times more movies than branded non-alcoholic beverages (33% vs. 2%; p = 0.0001), five times more movies than branded beer (25% vs. 5%; p = 0.0004), and in four times more movies than branded other alcoholic drinks (28% vs. 7%; p = 0.0006).

**Table 2:** Portrayals of branded soft drinks compared to other branded beverages for 95 movies, 1991–2000

Soft drinks versus...	Movies Portraying Specific Branded Beverages								
	
	Neither Beverage		Both Beverages		Comparison Beverage Only		Soft Drinks Only		p-value
		
	N	%	N	%	N	%	N	%	
Non-alcoholic beverages ^a^	62	65.3%	0	0%	2	2.1%	31	32.6%	.0001
Beer ^b^	59	62.1%	7	7.4%	5	5.3%	24	25.3%	.0004
Other alcoholic beverages ^c^	57	60.0%	4	4.2%	7	7.4%	27	28.4%	.0006

a. Non-alcoholic beverages are any non-alcoholic beverage that is not a soft drink, such as milk, juice, sports drinks, juice mixes.

b. Beer is any alcoholic beverage defined by the producer as beer.

c. Other alcoholic beverages are any alcoholic beverage not defined as beer.

This result is notable given that the relationship is reversed for unbranded beverages. Unbranded non-alcoholic drinks appeared in 20 times more movies than unbranded soft drinks (p = 0.0001). Unbranded beer appeared in movies two times more often than unbranded soft drinks (p = 0.028). Unbranded alcoholic drinks appeared 11 times more frequently than unbranded soft drinks (p = 0.0001).

For instances of actor-endorsement, more movies depict an actor consuming a branded soft drink than another branded beverage category (Table 3). Actor endorsement of branded soft drinks occurred in five times the number of movies compared to actor endorsement of other non-alcoholic drinks (p = 0.0126). The number of branded soft drink portrayals was not statistically different from those for branded beer and other non-alcoholic branded drinks.

**Table 3:** Endorsement of branded soft drinks compared to other branded beverages in 95 movies, 1991–2000

Actor endorsement (consumption) of soft drinks versus...	Movies Portraying Specific Branded Beverages								
	
	Neither Beverage		Both Beverages		Comparison Beverage Only		Soft Drinks Only		p-value
		
	N	%	N	%	N	%	N	%	
Non-alcoholic beverages ^a^	82	86.3%	0	0%	2	2.1%	11	11.6%	.0126
Beer^b^	79	83.2%	3	3.2%	5	5.3%	8	8.4%	.4054
Other alcoholic beverages ^c^	79	83.2%	2	2.1%	5	5.3%	9	9.5%	.2850

a. Non-alcoholic beverages are any non-alcoholic beverage that is not a soft drink, such as milk, juice, sports drinks, juice mixes.

b. Beer is any alcoholic beverage defined by the producer as beer.

c. Other alcoholic beverages are any alcoholic beverage not defined as beer.

Actors were more likely to consume unbranded beverages other than soft drinks. For instance, ten times the percentage of movies were actor endorsed for unbranded non-alcoholic beverages compared to unbranded soft drinks (44% vs. 4%, p < .0001). The same pattern is present for actor endorsement of unbranded alcohol compared to unbranded soft drinks (p = 0.0001).

### Worldwide distribution of American movies

An analysis of the revenues of America's top-grossing movies confirms that images of branded soft drinks in these movies are shown worldwide. Ticket sales outside of the U.S. for the 31 movies with depictions of branded soft drinks totaled $5 billion, or about 51% of total gross revenues. The proportion of non-U.S. revenue is similar to the entire sample of 95 movies, which earned 54% of ticket sales from countries outside of the U.S.

## Discussion

Branded soft drinks appeared in 72% of the movies where soft drinks were depicted, a proportion both statistically and substantially higher than any other beverage category including beer. Branded soft drinks also were significantly more likely to appear and to be actor-endorsed than other branded beverages. American movies earn about half of their movie theater revenues from sales outside the U.S., suggesting that images of branded soft drinks in movies can be a strategy for reaching a worldwide audience.

The finding that soft drinks are more likely to be seen in comedies was unexpected, and may be due to comedies being centered around social situations, many of which include eating and drinking. Unexpected was the absence of difference between the amount of time branded and unbranded soft drinks appeared on screen. This may indicate directors' sensitivity to their films being perceived as commercials. We also speculate that longer portrayals of branded products would only bring attention to those products, and raise flags for an audience already skeptical of advertising.

There may be several reasons why branded soft drinks were more likely to be portrayed and to be actor-endorsed compared to other branded beverages. For instance, beer companies may be sensitive to criticism of product placement in movies targeted at people under the legal drinking age. In 1999 the U.S. Federal Trade Commission chastised beer companies for paid product placements in PG and PG-13 films "with significant appeal to teens and children (including films with animal and "coming-of-age" themes); [and] in films for which the advertiser knew that the primary target market included a sizeable underage market[27]." In contrast, American soft drink makers engaged in rapid expansion of international markets during the 1990's, and sought numerous ways to create brand recognition, including product placement[28]. During this same period, product placement became an integral part of a larger marketing plan to reach American audiences[16].

Because product placement agreements are proprietary, we cannot verify that any particular depiction of a branded soft drink was the result of a paid agreement. Directors may choose to use branded products to make their movies more authentic or to convey certain attributes about the character. For instance, it was reported that neither AOL nor Federal Express paid for prominent roles in the movies *You've Got Mail *and *Castaway *respectively[29]. However, product placement has been widely acknowledged as an "essential" part of a wider product marketing strategy. This marketing strategy is illustrated in our finding that only one of 31 movies included portrayals of competing branded soft drinks.

## Conclusion

Until recently, the negative health consequences of soft drink consumption were limited to dental caries as identified in the current research. As soft drink consumption skyrocketed, the concern about soft drinks has shifted to obesity and its associated health consequences. Cross-sectional[30] and longitudinal studies[4,31] have linked soft drink consumption with obesity. Higher consumption also is associated with lower milk and higher total energy intakes[32]. For these reasons, health researchers and policy makers should consider product placement in movies and its potential influences on children.

### Future research: Monitoring product presence in movies

This study provides a useful baseline to continue monitoring the presence of branded soft drinks in popular movies. As pressure increases to limit aggressive marketing of soft drinks to children, we speculate that the soft drink industry may chose to increasingly rely on more subtle forms of advertising, including product placement in movies. Monitoring product placement of soft drinks in movies should continue in order to track any changes in product placement strategies and methods. For instance, a new technology allows the U.S. version of a movie to feature one branded product, while depicting another branded product in the movie's overseas distribution. The 2004 movie *Spider Man 2 *featured Dr. Pepper in its U.S. distribution, and the beverage Mirinda for the overseas audience[33].

### Policy options: What to do about product placement?

The soft drink industry's payment for product placement in movies is legal, although inherently misleading when the promotional intent of the placement is not revealed. Regardless of whether product placement affects health or not, the public has the right to know when advertising is occurring in movies. Disclosure requirements shown during the movie credits would be one way to inform viewers about the financial relationship between the soft drink companies and movie producers. This solution would have little effect, however, since few moviegoers stay until the end of the credits. A more effective disclosure strategy would be to flash "Product placement paid by [name of company]" on the movie screen each time a branded product appears. While a more effective warning, it is unlikely that movie producers or soft drink producers would agree to this disclosure strategy.

The Writers Guild and the Screen Actors Guild have called for a code of conduct that includes disclosure at the beginning of each movie when advertising has been incorporated into the script[34]. Disclosure at the beginning of each movie would inform the public that they will be subjected to advertising without interrupting the flow of the movie. It remains to be seen, however, whether voluntary disclosure would occur.

## Declaration of competing interests

The author(s) declare that they have no competing interests.

## Authors' contributions

Diana Cassady developed the major ideas for the paper and wrote the first draft. Marilyn Townsend contributed to the literature review and analysis strategy. Robert Bell developed and supervised the content analysis of the larger study of portrayals of food and physical activity in popular movies. Mitchell Watnik conducted the statistical analysis. All authors had critical input into the writing of the paper.
